# Constitutive and Inducible Resistance to Thrips Do Not Correlate With Differences in Trichome Density or Enzymatic-Related Defenses in Chrysanthemum

**DOI:** 10.1007/s10886-020-01222-1

**Published:** 2020-10-22

**Authors:** Gang Chen, Peter G. L. Klinkhamer, Rocío Escobar-Bravo

**Affiliations:** 1 Research Group Plant Ecology and Phytochemistry, Cluster Plant Sciences and Natural Products, Institute of Biology, Leiden University, Leiden, The Netherlands; 2 College of Forestry, Sichuan Agricultural University, Chengdu, China; 3 Present Address: Institute of Plant Sciences, University of Bern, Altenbergrain 21, 3013 Bern, Switzerland

**Keywords:** *Chrysanthemum* × *morifolium*, *Frankliniella occidentalis*, Jasmonic acid, Plant resistance, Polyphenol oxidase, Trichome density

## Abstract

Western flower thrips (WFT), *Frankliniella occidentalis*, is a serious insect pest of Chrysanthemum [*Chrysanthemum* × *morifolium* Ramat. (Asteraceae)]. Here we have investigated whether genotypic variation in constitutive and inducible resistance to WFT correlates with phenotypic differences in leaf trichome density and the activity of the defense-related enzyme polyphenol oxidase (PPO) in chrysanthemum. Non-glandular and glandular leaf trichome densities significantly varied among ninety-five chrysanthemum cultivars. Additional analyses in a subset of these cultivars, differing in leaf trichome density, revealed significant variation in PPO activities and resistance to WFT as well. Constitutive levels of trichome densities and PPO activity, however, did not correlate with chrysanthemum resistance to WFT. Further tests showed that exogenous application of the phytohormone jasmonic acid (JA) increased non-glandular trichome densities, PPO activity and chrysanthemum resistance to WFT, and that these effects were cultivar dependent. In addition, no tradeoff between constitutive and inducible resistance to WFT was observed. JA-mediated induction of WFT resistance, however, did not correlate with changes in leaf trichome densities nor PPO activity levels. Taken together, our results suggest that chrysanthemum can display both high levels of constitutive and inducible resistance to WFT, and that leaf trichome density and PPO activity may not play a relevant role in chrysanthemum defenses against WFT.

**Electronic supplementary material:**

The online version of this article (10.1007/s10886-020-01222-1) contains supplementary material, which is available to authorized users.

## Introduction

Plants have evolved sophisticated constitutive and inducible defenses to defend themselves against arthropod herbivores. These defenses can be physical structures (e.g., trichomes, thorns, spines) and/or chemical components (e.g., secondary metabolites and defense-related enzymes) present in the plant prior to herbivory (Rosner and Hannrup 2004; Franceschi et al. 2005; Mithöfer and Boland 2012) or specifically induced upon herbivore attack (Erb et al. 2012). Both the expression of constitutive and inducible defenses can vary within and among plant species (Underwood et al. 2000; Koornneef et al. 2004; Zhang et al. 2020). Identification and characterization of novel sources of pest resistance is therefore a fundamental step to transfer these defenses into susceptible plant varieties through plant breeding programs (Macel et al. 2019; Visschers et al. 2019).

Chrysanthemum [*Chrysanthemum* × *morifolium* Ramat. (Asteraceae)] is one of the economically most important ornamental crops worldwide (Fletcher 1992; Steenbergen et al. 2018) but it is highly susceptible to multitude of arthropod pests. Western flower thrips (WFT) *Frankliniella occidentalis* [Pergande] is one of the major insect pests of chrysanthemum, severely affecting its marketing quality (Mouden et al. 2017; Reitz et al. 2020). WFT feeding on leaves, flowers and fruits can reduce plant photosynthetic capacity, growth and reproduction (de Jager et al. 1995a, b). Adults and larvae insert their stylets into the plant tissue and ingest cell contents while feeding. This causes the cells to collapse or fill with air, giving the thrips-feeding area in the plant a silvery appearance called “silver damage” (Steenbergen et al. 2018). In addition, WFT can transmit devastating virus diseases (Maris et al. 2003). Currently, the use of insecticides has been the most common strategy for WFT control, which entails serious risks for the environment and human health. Alternative agricultural practices such as the use of biological control agents and/or plant genotypes harboring insect-resistant traits constitute more environmentally friendly strategies for thrips control (Muñoz-Cárdenas et al. 2017; Bac-Molenaar et al. 2019).

Host plant resistance to WFT has been associated to the presence of both chemical and morphological defenses in chrysanthemum (De Jager et al. 1996). For instance, previous work by Leiss et al. (2009b) reported that leaf chlorogenic acid was positively associated to thrips resistance. Disruption of plant tissues by herbivory triggers the oxidation of chlorogenic acid by plant polyphenol oxidases (PPOs) and peroxidases (Duffey and Stout 1996). This may result in the production of highly reactive quinones that could inhibit the digestion of plant proteins by arthropod herbivores (Stout et al. 1994; War et al. 2012). In line with this, higher activities of PPO have also been associated to increased plant resistance to diverse arthropod herbivores (Mahanil et al. 2008; Bhonwong et al. 2009). In chrysanthemum, enhanced constitutive levels of PPO activity was reported in an aphid-resistant cultivar, suggesting a possible role of this defensive enzyme in chrysanthemum resistance to herbivory (He et al. 2011).

In addition to chemical defenses, density of non-glandular leaf trichomes and the gland size of leaf glandular trichomes have been reported to positively correlate with aphid resistance in chrysanthemum (He et al. 2011). Trichomes are hairy epidermal structures, mainly found in leaves and stems, that can be classified as non-glandular and glandular (Glas et al. 2012). Non-glandular trichomes function as physical hurdles, hindering the ability of insects to access the leaf surface and thus to feed and/or oviposit (Tian et al. 2012). Glandular trichomes also provide a physical barrier in plants, but they can chemically repel or poison arthropod herbivores as well (Kang et al. 2010; Bleeker et al. 2012). For instance, enhanced expression of leaf glandular trichomes density and chemistry increased tomato resistance against WFT in cultivated tomato (Escobar-Bravo et al. 2018). Yet, whether leaf trichomes play a relevant role in chrysanthemum defenses against thrips has not been elucidated.

Both trichome density and PPO activity are constitutively expressed in plants, but their expression can be modulated by abiotic and biotic factors (Biesiada and Tomczak 2012; Escobar-Bravo et al. 2017; Escobar-Bravo et al. 2019; Hauser 2014), as well as by defense elicitors. For instance, application of the phytohormone jasmonic acid (JA) has been reported to induce increased trichome densities in tomato (Boughton et al. 2005; Escobar-Bravo et al. 2017) and *Arabidopsis* (Traw and Bergelson 2003) among other plant species. Similarly, JA can also induce PPO in diverse plant species (Chen et al. 2018; Constabel and Ryan 1998; Thaler et al. 1996). Genotypic variation in plant responses to defense elicitors and/or herbivory can determine the expression of defense-related traits and, ultimately, resistance to herbivores (Sauge et al. 2006; Snoeren et al. 2010; Tu et al. 2018).

In this study, we investigated whether constitutive and JA-inducible chrysanthemum resistance to WFT correlates with differences in non-glandular and glandular leaf trichome densities and PPO activity levels. For this, we first explored the genotypic variation in leaf trichome densities among ninety-five chrysanthemum cultivars. Twelve of these cultivars differing in leaf trichome densities and PPO activities were used to determine whether these leaf traits correlated with WFT resistance by using non-choice whole plant insect bioassays. Finally, we determined variations in JA-mediated induction of plant defenses against WFT among chrysanthemum cultivars and tested whether the variation was explained by hormone-induced changes in trichome densities and PPO-associated defenses.

## Methods and Materials

### Plants and Insects

Ninety-five different chrysanthemum cultivars [provided by Dekker Chrysanten (Hensbroek), Deliflor Chrysanten (Maasdijk) and Dümmen Orange (De Lier)] were used in our study (Table S1). Chrysanthemum cuttings were first individually planted in plastic trays (4 cm × 4 cm × 6 cm) filled with potting soil. At 14 d after planting, plants were transplanted to plastic pots (9 cm × 9 cm × 10 cm) containing the same potting soil and randomly placed in a climate room provided with 20 °C, 70% RH, 113.6 μmol photons m^−2^ s^−1^ of photosynthetically active radiation (PAR) and L16:D8 photoperiod.

Western flower thrips (WFT), *Frankliniella occidentalis* (Pergande), were obtained from a colony reared on chrysanthemum flowers (cultivar ‘Euro Sunny’) in a climate room at 23 °C, 60% RH and L12:D12 photoperiod.

### Experimental Design

To investigate phenotypic variation in constitutive and inducible chrysanthemum defenses associated to WFT resistance, we performed three different experiments.

First, we determined constitutive levels of non-glandular and glandular trichome densities on leaves of ninety-five chrysanthemum cultivars at 35 d after planting (Experiment 1) as described below.

Second, we selected twelve of the ninety-five cultivars that displayed contrasting levels of trichome densities to further determine whether constitutive levels of trichome density correlated with WFT resistance (Experiment 2). This set of plants was further used to test differences in PPO activity levels. Plants were sampled for determination of non-glandular and glandular trichome density and PPO activity, or used for non-choice whole plant thrips bioassays, at 35 d after planting as described below.

Third, we tested whether application of the phytohormone jasmonic acid (JA) enhanced WFT resistance in six cultivars, and whether JA induces trichome- and PPO-associated defenses (Experiment 3). Due to limitations in plant material availability, only two genotypes previously tested for WFT resistance in Experiment 2 (#33 and 34) were used for this experiment, while the other four genotypes were selected from the ninety-five chrysanthemum varieties used in Experiment 1. This selection was based on trichome density variations and thrips susceptibility levels provided by the breeding companies. For this experiment, chrysanthemum plants were sprayed with approximately 5 ml of 3 mM JA (Cayman, Ann Arbor, Michigan, USA) in 2.4% aqueous ethanol solution as described by Redman et al. (2001). Control plants were sprayed with a similar volume of a mock solution consisting of 2.4% aqueous ethanol. Seven days after the hormone treatment, mock- and JA-treated plants were sampled for determination of non-glandular and glandular trichome density, PPO activity, or used for non-choice whole plant thrips bioassays as detailed below. We selected this sampling time because previous studies in tomato and *Arabidopsis* have determined that a significant increment of trichome density can be observed 7 days after the hormone application (Boughton et al. 2005; Yoshida et al. 2009). In addition, higher PPO activities can be detected up to 7 days after JA induction in tomato (Thaler et al. 2001).

### Analysis of Trichome Density and Morphology

In all the experiments, densities of glandular and non-glandular trichomes were determined on the adaxial leaf surface of the third leaf from the apex (3, 5 and 7 biological replicates for Experiment 1, 2 and 3, respectively). This leaf was chosen because previous studies in chrysanthemum and other plant species have shown that younger leaves display higher constitutive and inducible levels of trichome densities (Stavrinides and Skirvin 2003; Chen et al. 2018). Two pictures were taken in the middle part of the leaf at both sides of the main vein, each covering an area of 12 mm^2^, using a stereomicroscope (MZ16, Leica Microsystems, Wetzlar, Germany). Trichome number was counted in both pictures using the software 64-bit Fiji ImageJ (http://fiji.sc/Fiji), and the average of the two measurements was expressed as number of trichomes per cm^2^.

### Scanning Electron Microscopy (SEM)

SEM analysis was conducted on the adaxial side of the third leaf from the apex. Leaves were fixed in 2.5% (*v*/v) glutaraldehyde in 0.1 M phosphate buffer (PBS) (pH 7.2) at room temperature. Samples were then dehydrated in acetone series of 50, 70, 90, 96, and 100% (v/v) and dried in a Bal-Tec CPD 030 Critical Point Dryer with liquid CO_2_ (Leica Microsystems). The samples were coated with gold in a Polaron SEM coating unit E5100. SEM images were taken with a JEOL 6400 scanning electron microscope at the Microscopy Unit of the Institute of Leiden (The Netherlands).

### Determination of PPO Activity

PPO activity was determined in the third leaf from the bottom following the methodology described in Stout et al. (1998). This leaf was chosen based on previous work that showed a stronger inducibility of PPO levels in basal chrysanthemum leaves (Chen et al. 2020). Briefly, 0.150 g of leaf tissue without midrib was flash frozen in liquid nitrogen, ground with a tissue-lyser (Qiagen, Hilden, Germany) and homogenized in a 2 ml tube with 1.25 ml ice-cold 0.1 M pH 7.0 phosphate buffer containing 7% polyvinylpolypyrolidine and 0.4 ml of 10% Triton X-100. The homogenate was vortexed for 2 min and centrifuged for 10 min at 11,000×g and 4 °C. Five microliters of the supernatant was added to 1 ml of 2.92 mM chlorogenic acid solution in pH 8.0 phosphate buffer. The optical density (OD) at 470 nm was recorded in a spectrophotometer (UV-1800, Shimadzu) every 10 sec for 1 min. PPO activity was defined as the increment of OD values per min per gram of fresh weight.

### Non-Choice Whole Plant Thrips Bioassay

Non-choice whole-plant bioassays were performed as described by Leiss et al. (2009a). For this, individual plants (10 and 7 replicates for Experiment 2 and 3, respectively) were placed into WFT-proof cages consisting of Perspex plastic cylinders (50 cm height and 20 cm diameter) closed at the top with a displaceable ring of nylon gauze (120 μm pore mesh size). Ten adult WFT (8 females and 2 males) were added to each plant. Plants were maintained in a climate room provided with 113.6 μmol photons m^−2^ s^−1^ of PAR, 16 L:8D of photoperiod, 25 °C and 70% RH. WFT feeding damage (for examples see Steenbergen et al. 2018), hereafter referred to as ‘silver damage’, was visually scored for each leaf of the plant using a grid to measure the damaged area in mm^2^, at 7 days after infestation. Whole plant silver damage was calculated by adding up the damage of each individual leaf.

### Statistical Analysis

Normality and homogeneity of residuals were checked using Kolmogorov-Smirnov and Levene’s tests, respectively. We used one-way ANOVA to test for significant differences in non-glandular trichome densities (Experiment 1) and PPO activity (Experiment 2) among cultivars. Data were square root transformed for non-glandular trichome density to meet the requirements of the ANOVA. Differences in glandular trichome density (Experiment 1) and silver damage symptoms (Experiment 2) among cultivars were analyzed by Kruskal-Wallis test. As a measure for the phenotypic variation across cultivars, the percent coefficient of variation (CV) was calculated. CV is expressed as the ratio of the standard deviation (δ) to the mean (μ), i.e. % CV = δ/μ ·100 (Sokal and Rohlf 1995). A high CV value means high phenotypic variation for the studied trait. The relationships between (1) non-glandular and glandular trichome densities (Experiment 1), (2) silver damage and PPO activity or trichome density (Experiment 2), and (3) constitutive and induced resistance indexes (Experiment 3) were determined by Pearson or Spearman correlation tests. In Experiment 3, the effects of the hormone treatment, plant genotype and their interaction on silver damage, PPO activity and glandular trichome density were analyzed by Generalized Linear Models (GLM) by using linear distribution and identity link functions. Differences among groups were tested by Fisher’s least significant difference (LSD) post-hoc test. The constitutive resistance index (CRI) was calculated for each cultivar in Experiment 3 by dividing the silver damage symptoms by the silver damage symptoms of a “reference cultivar”. The “reference cultivar” was selected based on the lowest silver damage symptoms and, therefore, the highest constitutive resistance index, set as 1. The induced resistance index (IRI) was calculated for a given cultivar as the percent reduction in silver damage symptoms in JA-treated plants respect to controls: [(average of silver damage detected on JA-treated plants – average silver damage detected on mock-treated plants) / average of silver damage on mock-treated plants] (Brody and Karban 1992). Statistical analyses were performed by using the SPSS software package (version 25; SPSS Inc., Chicago, IL, USA). All detailed statistics are included in Table S2.

## Results

### Non-glandular and Glandular Trichome Densities Vary among Chrysanthemum Cultivars

**Fig. 1:**
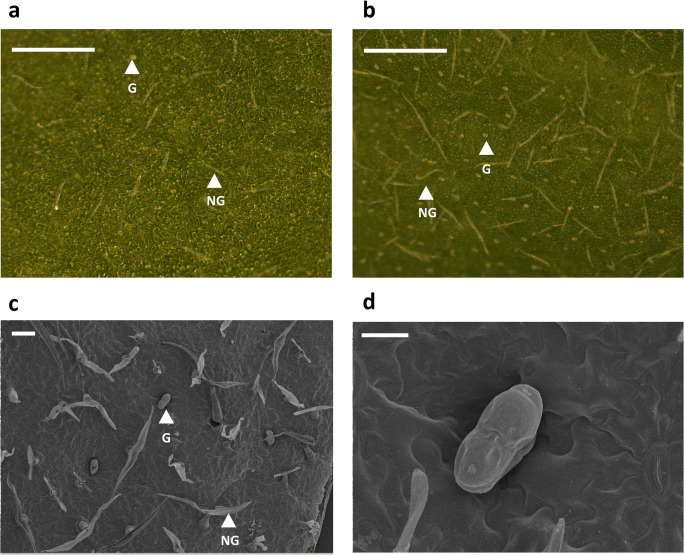
Representative micrographs of non-glandular and glandular trichomes on chrysanthemum leaves. Light micrographs of the adaxial leaf surface of a chrysanthemum cultivar displaying (**a**) low and (**b**) high trichome densities. Scanning electron microscopic images of the adaxial leaf surface of chrysanthemum leaves (**c** and **d**). Bean-shape glandular trichomes are shown in (**d**). White arrows indicate the position of non-glandular (NG) and glandular (G) trichomes. The white bars represent 100 μm in (**a**), (**b**) and (**c**), and 30 μm in (**d**)

The morphological analysis of chrysanthemum leaves revealed the presence of two types of trichomes, non-glandular and glandular, the latter having a bean-shape structure and coinciding with the description reported by He et al. (2011) (Fig. 1).

**Fig. 2:**
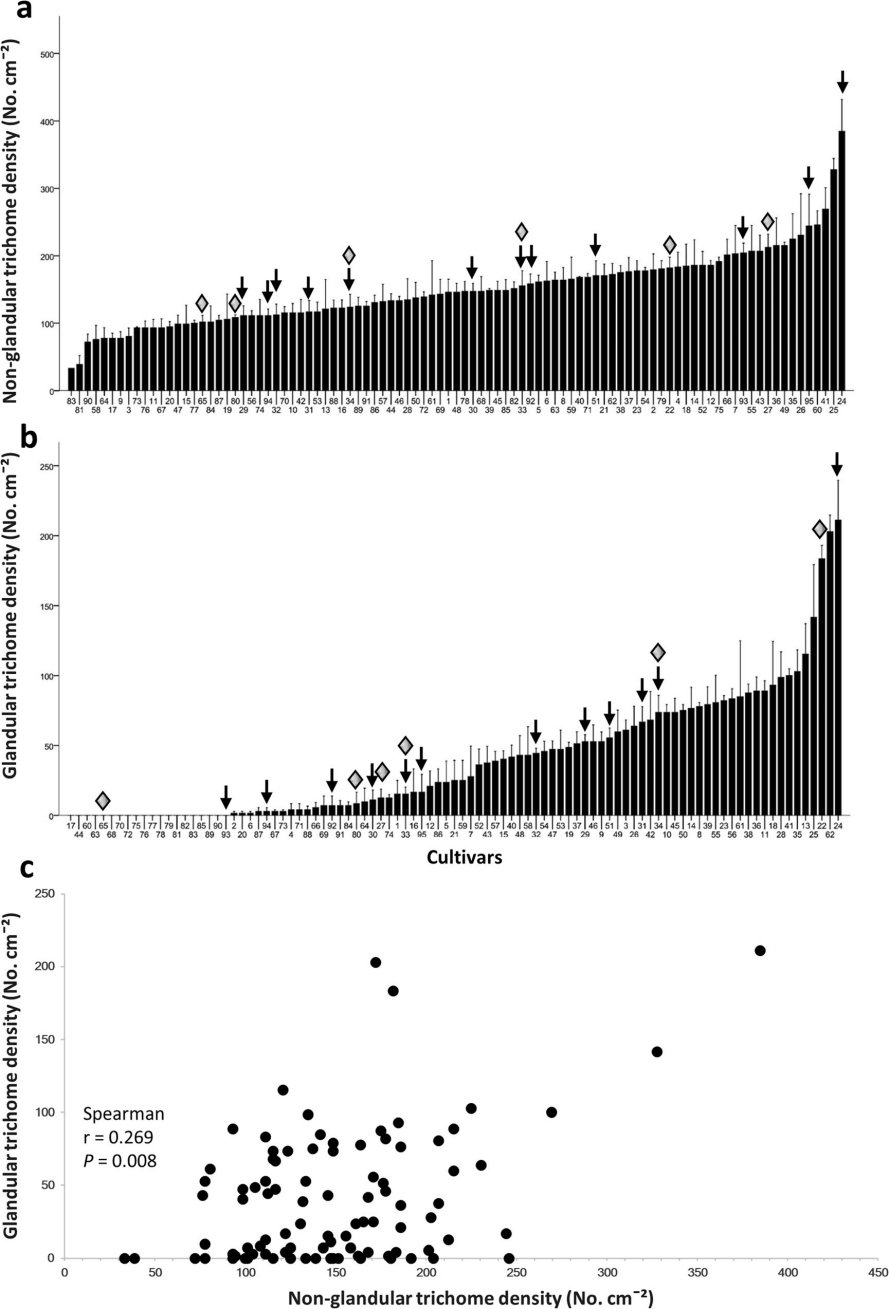
Genotypic variation in non-glandular and glandular trichome densities. Bars depict the mean (± SEM, *n* = 3) of leaf (**a**) non-glandular and (**b**) glandular trichome densities analyzed in 95 chrysanthemum cultivars at 35 days after planting. Trichome density was determined on the adaxial side of the third leaf from the apex. The arrows indicate the twelve cultivars used in Experiment 2, while the diamonds indicate the six cultivars used in Experiment 3. (**c**) Scatter plot depicting the relationship between non-glandular and glandular trichome densities. Each dot corresponds to the mean of three plant replicates per cultivar

The density of non-glandular trichomes significantly differed among cultivars (ANOVA, *F*_94, 190_ = 6.595, *P* < 0.001), ranging from 33 trichomes/cm^2^ (cultivar 83) to 384 trichomes/cm^2^ (cultivar 24) (Fig. 2a). The CV of non-glandular trichomes density was 37%. Similarly, glandular trichome densities varied among cultivars (Kruskal-Wallis, *H* = 250.077, *df* = 94, *P* < 0.001), with a CV of 113% (Fig. 2b). Out of the ninety-five cultivars analyzed, twenty of them had no glandular trichomes, and the highest density of glandular trichomes was 211 trichomes/cm^2^ (cultivar 24). In addition, densities of non-glandular and glandular trichomes were positively correlated (Fig. 2c; two-tailed Spearman, *r* = 0.269, *N* = 95, *P* = 0.008).

### Trichome Density and PPO Levels Do Not Correlate with Chrysanthemum Resistance to WFT

**Fig. 3:**
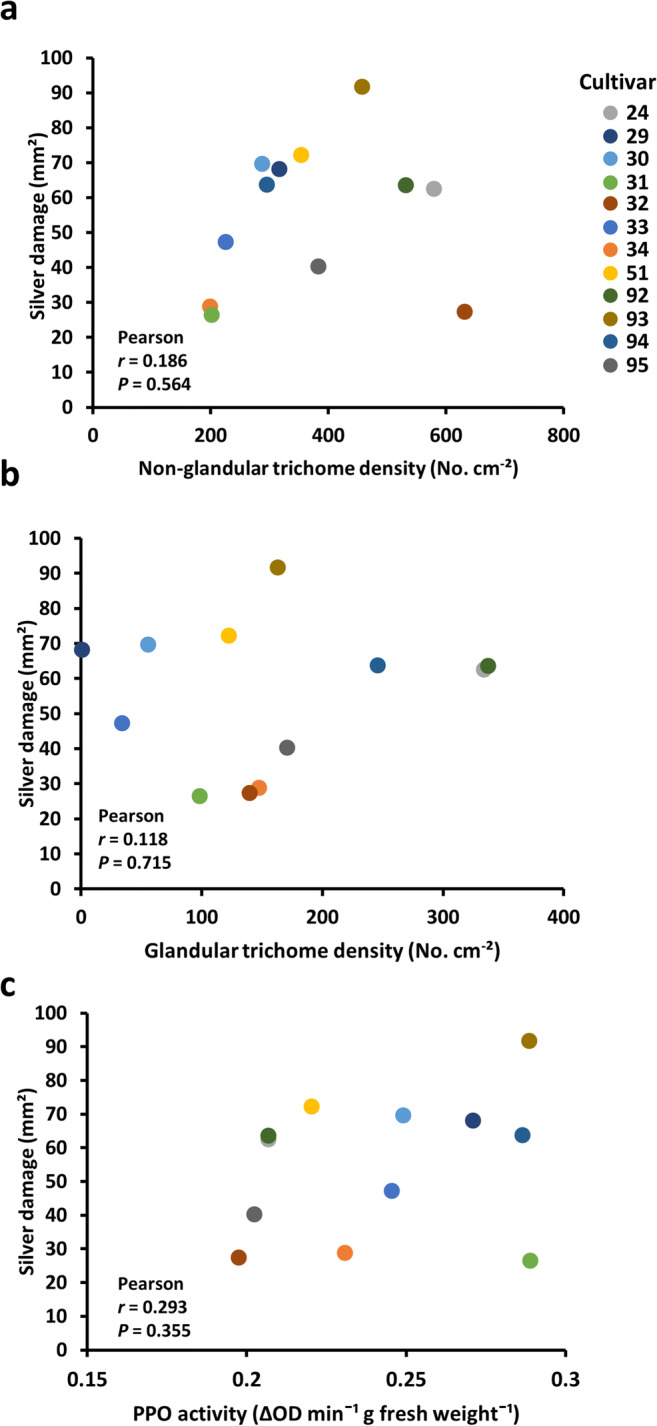
Relationship between Western flower thrips resistance and putative defense-related traits in chrysanthemum. Scatter plots depicting the relationship between (**a**) silver damage and non-glandular trichome density, (**b**) silver damage and glandular trichome density and (**c**) silver damage and polyphenol oxidase (PPO) activity levels. Plants were sampled for PPO activity and trichome density, or subjected to non-choice whole plant thrips bioassays, at 35 days after planting. Silver damage symptoms were evaluated at 7 days after thrips infestation. The plots display data obtained from 12 chrysanthemum cultivars. Each dot corresponds to the mean of five plant replicates per cultivar for PPO and trichome density, and of ten plant replicates per cultivar for silver damage symptoms

To determine whether trichome density correlated with chrysanthemum resistance to WFT, we selected twelve chrysanthemum cultivars differing in trichome densities (Fig. 2) to test for WFT resistance in non-choice whole plant bioassays. Silver damage symptoms significantly differed among the cultivars with a CV of 38% (Fig. S1a; Kruskal-Wallis test, *H* = 48.831, *df* = 11, *P* < 0.001). However, no significant correlations between silver damage and non-glandular (Fig. 3a; two-tailed Pearson, *r* = 0.186, *N* = 12, *P* = 0.564) or glandular trichome densities (Fig. 3b; two-tailed Pearson, *r* = 0.118, *N* = 12, *P* = 0.715) were observed.

To test whether variation in WFT susceptibility correlated with differences in leaf chemical defenses, we determined constitutive levels of PPO activity in the selected twelve cultivars (Fig. 3c). PPO activity significantly differed among the chrysanthemum cultivars (Fig. S1b; ANOVA, *F*_11, 48_ = 6.595, *P* = 0.002), but the CV (15%) was lower than that of silver damage (38%). In addition, PPO activity did not correlate with glandular trichome densities (Fig. S2a; two-tailed Pearson, *r* = −0.376, *N* = 12, *P* = 0.228) or with non-glandular trichome densities (Fig. S2b; two-tailed Pearson, *r* = −0.567, *N* = 12, *P* = 0.055). Furthermore, PPO activity did not correlate with thrips-associated leaf damage (Fig. 3c**;** two-tailed Pearson, *r* = 0.293, *N* = 12, *P* = 0.355).

### Induction of Chrysanthemum Resistance to WFT Differs among Chrysanthemum Cultivars and Is Not Explained by Variations in PPO and Trichome Density

**Fig. 4:**
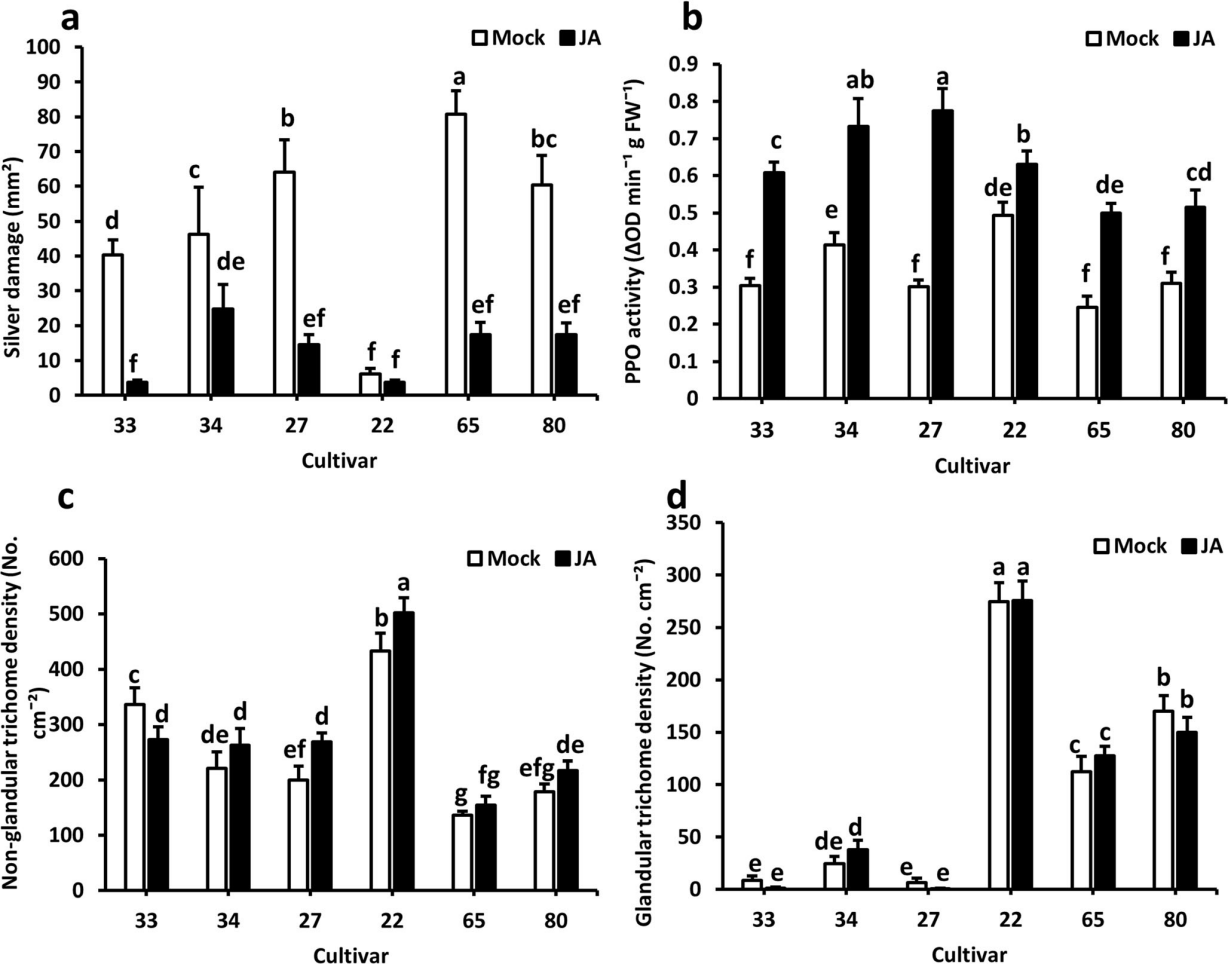
Genotypic variation in jasmonic acid-mediated induction of Western flower thrips resistance among chrysanthemum cultivars. Six different chrysanthemum cultivars were treated with mock or jasmonic acid (JA) solutions at 28 d after planting. Plants were sampled for determination of PPO activity and trichome density or subjected to non-choice whole plant bioassays at 7 days after the hormone treatments. (**a**) Silver damage symptoms (mean ± SEM, *n* = 7) evaluated 7 days after thrips infestation. (**b**) PPO activity, (**c**) non-glandular trichome density and (**d**) glandular trichome density (mean ± SEM, *n* = 5–7). Different letters indicate significant differences among groups compared by Fisher’s least significant differences (LSD) test at *P* ≤ 0.05

Constitutive levels of non-glandular and glandular trichome densities and PPO activity were not correlated with chrysanthemum resistance to WFT. Thus, we further explored whether differences in the inducibility of these defenses could explain the variation in WFT susceptibility among chrysanthemum cultivars. For this, we selected six cultivars differing in trichome densities (Fig. 2) and thrips susceptibility (data not shown). Silver damage symptoms significantly differed among the cultivars (Fig. 4a; GLM, *χ*^2^ = 70.979, *df* = 5, *P* < 0.001 for cultivar). Application of JA significantly reduced silver damage symptoms (GLM, *χ*^2^ = 111.912, *df* = 1, *P* < 0.001 for JA application) and this effect depended on the chrysanthemum cultivar (GLM, *χ*^2^ = 33.160, *df* = 5, *P* < 0.001 for the interaction between JA treatment and cultivar).

JA induced PPO activity in all the chrysanthemum cultivars (Fig. 4b; GLM, *χ*^2^ = 58.106, *df* = 5, *P* < 0.001 for JA treatment), and this induction was also dependent on the cultivar (GLM, *χ*^2^ = 25.740, *df* = 5, *P* < 0.001 for the interaction between JA treatment and cultivar). Non-glandular trichome density varied among cultivars (GLM, *χ*^2^ = 261.895, *df* = 5, *P* < 0.001 for cultivar) (Fig. 4c), and it was significantly induced by JA (GLM, *χ*^2^ = 5.034, *df* = 1, *P* = 0.025 for JA treatment) depending on the cultivar (GLM, *χ*^2^ = 12.661, *df* = 5, *P* = 0.027 for the interaction between JA treatment and cultivar). Glandular trichome density varied among cultivars as well (Fig. 4d, GLM, *χ*^2^ = 1036.121, *df* = 5, *P* < 0.001 for cultivar). However, JA did not increase glandular trichome densities in any chrysanthemum cultivars (GLM, *χ*^2^ = 0.010, *df* = 1, *P* = 0.922 for JA treatment; *χ*^2^ = 4.057, *df* = 5, *P* = 0.541 for the interaction between JA treatment and cultivar).

**Fig. 5:**
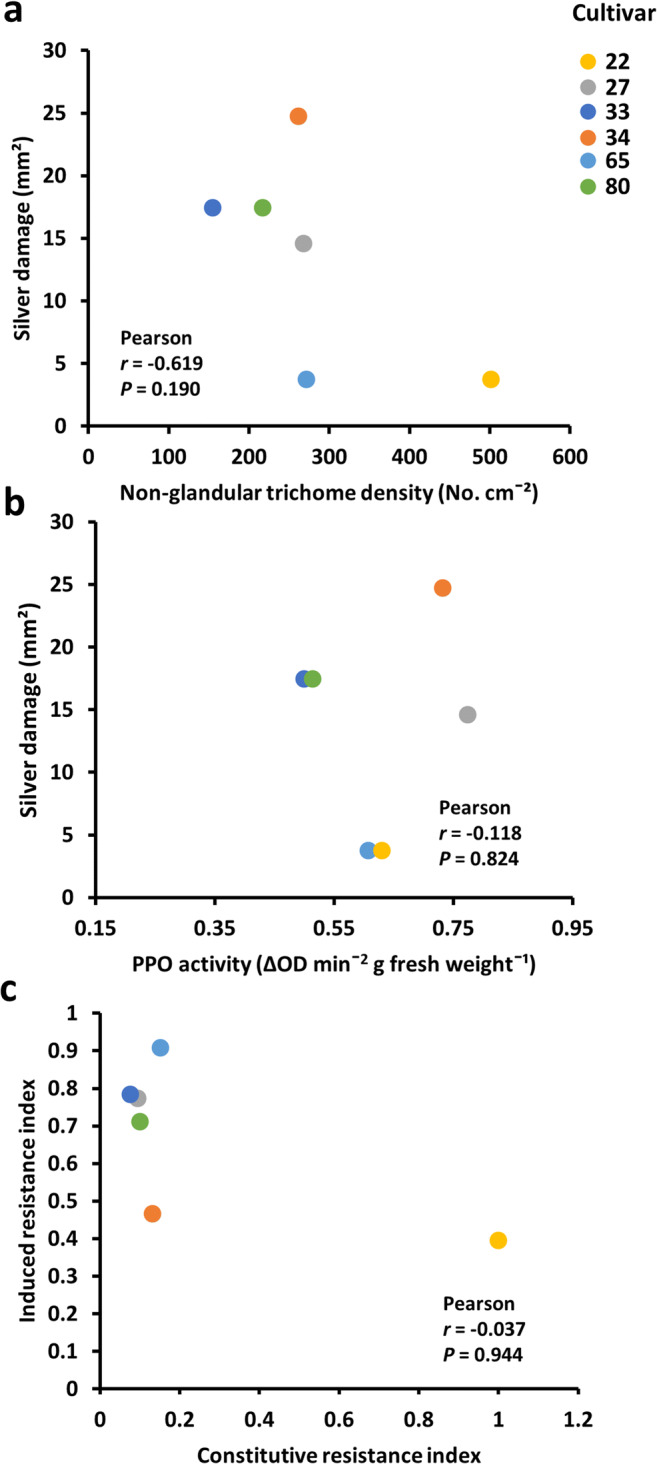
Relationship between JA-associated induced defenses and chrysanthemum resistance to Western flower thrips**.** Plants were treated with a JA solution at 28 days after planting and sampled for PPO activity, trichome density or subjected to non-choice whole plant thrips bioassays at 7 days after the hormone treatments. Silver damage symptoms were evaluated at 7 days after thrips infestation. Scatter plots depicting: (**a**) the relationship between silver damage and non-glandular trichome density, and (**b**) silver damage and polyphenol oxidase (PPO) activity in jasmonic acid (JA)-treated plants corresponding to six different chrysanthemum cultivars. Each dot corresponds to the mean of five plant replicates per cultivar for PPO activity, and of seven plant replicates per cultivar for silver damage symptoms. (**c**) Relationship between constitutive resistance index (CRI) and induced resistance index (IRI)

The density of non-glandular trichomes (Fig. 5a; two-tailed Pearson, *r* = −0.691, *N* = 6, *P* = 0.190) and PPO activity (Fig. 5b; two-tailed Pearson, *r* = 0.118, *N* = 6, *P* = 0.824) did not correlate with silver damage symptoms in JA-treated plants. Finally, CRI did not correlate with IRI for these six cultivars (Fig. 5c; two-tailed Pearson, *r* = −0.037, *N* = 6, *P* = 0.944).

## Discussion

Our study has demonstrated that there is great variation in both constitutive and JA-inducible resistance to Western flower thrips (WFT) among chrysanthemum cultivars, thus opening new avenues for pest-resistance breeding in this horticultural species. Yet, we found that this variation could not be explained by differences in leaf trichome densities or polyphenol oxidase activity, which are relevant plant defenses in other plant-herbivores interactions systems.

Several studies have reported the presence of trichomes in chrysanthemum leaves (Cheng et al. 2011; He et al. 2011; Stavrinides and Skirvin 2003; Sun et al. 2013). These reports, however, characterized only two or three chrysanthemum genotypes. Our results showed significant phenotypic variation in non-glandular and glandular leaf trichome densities in a larger sample of chrysanthemum cultivars. These differences did not correlate with WFT resistance, which contrasts with the defensive role of leaf trichomes against WFT demonstrated in tomato (*Solanum lycopersicum*) (Escobar-Bravo et al. 2018, 2019). In chrysanthemum, a higher density of non-glandular trichomes has been previously observed in aphid-resistant cultivars (Cheng et al. 2011; He et al. 2011), which suggests that trichome-mediated effects on herbivore performance may depend on the herbivore species. Maharijaya et al. (2015) found no correlation between leaf trichome densities and WFT resistance in pepper (*Capsicum annuum*), whereas Yadwad et al. (2008) described resistance levels against another thrips species, *Scirtothrips dorsalis*, in hairy genotypes of pepper*.*

Differences in the profiles and/or abundance of glandular trichome-derived allelochemicals can determine the levels of plant resistance to arthropod herbivores (Antonious et al. 2005). The chemical composition of leaf glandular trichomes has not been previously reported in chrysanthemum, nor was it characterized in our study. We do not rule out the possibility that differences in the production of trichome-derived allelochemicals, rather than the density, could play a role in chrysanthemum resistance to WFT (Bac-Molenaar et al. 2019). Interestingly, He et al. (2011) reported larger glands for leaf trichomes in an aphid-resistant chrysanthemum genotype compared to gland size in a susceptible genotype, which could be explained by a higher production and storage of trichome-derived chemicals. Additional studies are thus needed to determine (1) whether glandular trichomes in chrysanthemum are biochemically active, (2) the identity of the compounds they produce, and (3) whether these putative compounds confer anti-herbivory properties and explain differences in WFT susceptibility among cultivars.

Our study showed that there was a significant phenotypic variation in the activity of the defensive protein PPO among chrysanthemum cultivars. PPO activity has been reported to correlate with plant resistance to herbivorous insects (Bhattacharya et al. 2009; Wei et al. 2007). In our study, however, the activity of this enzyme was not associated with WFT resistance. We note that selection of the twelve genotypes to test WFT susceptibility was initially based on variation in trichome densities, leading us to speculate that we did not assess the whole spectrum of PPO expression among cultivars. Alternatively, the lack of correlation of PPO with chrysanthemum resistance to WFT may be explained by the absence of specific enzymatic substrates this protein can interact with. For instance, constitutive levels of chlorogenic acid, one of the main enzymatic substrates of PPO, have been shown to strongly differ among chrysanthemum genotypes and positively correlate with resistance to WFT (Leiss et al. 2009b). Thus, we hypothesize that variations in foliar chlorogenic acid levels might determine the effectiveness of PPO-associated defenses. Additional correlational analysis between chlorogenic acid and PPO activity levels on a larger number of genotypes should be performed in future studies.

Activation of JA signaling has been reported to enhance plant resistance to WFT in some plant species (Abe et al. 2009; Chen et al. 2018; Escobar-Bravo et al. 2017; Li et al. 2002), including chrysanthemum (Chen et al. 2020). Our study further reveals that the effect of JA on chrysanthemum resistance to WFT is genotype-dependent (Fig. 4a). Genotypic variation in the induction of plant defenses within plant species has been amply reported (Agrawal 1999; Brody and Karban 1992; English-Loeb et al. 1998; Sauge et al. 2006; Underwood et al. 2000; Zhang et al. 2020). This has been attributed to differences in the chemical profiles and/or in the presence of potentially inducible defense traits among genotypes within a plant species. As leaf trichomes and PPO activities can be induced by either JA or thrips infestation (Escobar-Bravo et al. 2017), we tested whether a differential induction of these defenses might explain the variation in thrips susceptibility among chrysanthemum cultivars. Our results showed that, despite the positive effect of JA on non-glandular trichome density and PPO activity, the induction of these defenses could not account for resistance to WFT. Surprisingly, our results also showed that JA did not increase glandular trichome density, which contrasts with its reported induction in other plant species (Boughton et al. 2005; Escobar-Bravo et al. 2017). Hormonal induction of leaf glandular trichomes might be therefore developmentally and/or environmentally dependent in chrysanthemum (Hare and Walling 2006; Xue et al. 2019). Alternatively, glandular trichome production in chrysanthemum may need more than 7 days to respond to the hormone treatment. In other studies, the time required for changes in leaf trichome densities after herbivory or JA application ranged from days to weeks (Dalin et al. 2008).

Taken together, the chemical or physical traits that contribute to JA-mediated induction of chrysanthemum resistance to WFT are still unknown. However, in a recent study, variations in JA-mediated induction of sugars and amino acids, as well as of defensive chemicals such as chlorogenic acid, coincided with differences in thrips susceptibility between young and old leaves of chrysanthemum plants (Chen et al. 2020). These compounds could be targets for future studies. In peppers, QTL mapping has recently been used to identify a genetic locus responsible for about 50% of the resistance of this species to thrips (Maharijaya et al. 2015), and this approach could be used to generate hypotheses about the basis for resistance in chrysanthemums as well.

Expression of constitutive and inducible defenses are often not independent of each other, and many studies have shown that constitutive defenses negatively correlate with induced plant defenses (e.g. Herms and Mattson 1992; Rasmann et al. 2015). However, tradeoff between constitutive and inducible resistance has been found to be more significant among wild plant species than among cultivated species (Kempel et al. 2011). In line with this, we showed that JA-mediated induction of chrysanthemum resistance to WFT did not correlate with constitutive defenses (Fig. 5c). Indeed, one of the chrysanthemum cultivars displayed both the lowest silver damage symptoms under control conditions (Fig. 4a; genotype 33) and the strongest reduction in WFT-associated damage after JA application (tenfold reduction) than the most susceptible cultivars. These findings also agree with previous reports in other cultivated species (Zhang et al. 2009). For instance, plant constitutive resistance to spider mites (*Tetranychus turkestan*) and the Mexican bean beetles (*Epilachna varivestis*) did not correlate with herbivory-induced resistance in cotton (*G. hirsutum*) and soybean (*Glycine max*), respectively (Brody and Karban 1992; Underwood et al. 2000). Our results have important implications for plant breeding purposes, as the lack of correlation between induced and constitutive resistance in chrysanthemum suggests the possibility to breed for cultivars with high levels of both types of resistance. For this, additional research will be needed to determine the chemical nature of both forms of resistance in chrysanthemum.

## Electronic supplementary material

ESM 1 (PDF 347 kb)
