# Supplementary material for: Constitutive and Inducible Resistance to Thrips Do Not Correlate With Differences in Trichome Density or Enzymatic-Related Defenses in Chrysanthemum
Source: J Chem Ecol. 2020 Oct 22;46(11):1105–16. doi: 10.1007/s10886-020-01222-1 (PMC7677159; doi:10.1007/s10886-020-01222-1)
Supplement: Supplementary file 1 — (PDF 347 kb) [file 10886_2020_1222_MOESM1_ESM.pdf]

CONSTITUTIVE AND INDUCIBLE RESISTANCE TO THRIPS DO NOT CORRELATE WITH DIFFERENCES IN TRICHOME DENSITY OR ENZYMATIC-RELATED DEFENSES IN CHRYSANTHEMUM

GANG CHEN *

Research group Plant Ecology and Phytochemistry, Cluster Plant Sciences and Natural Products, Institute of Biology, Leiden University, The Netherlands

College of Forestry, Sichuan Agricultural University, Chengdu, China

PETER G.L. KLINKHAMER ‡

Research group Plant Ecology and Phytochemistry, Cluster Plant Sciences and Natural Products, Institute of Biology, Leiden University, The Netherlands

ROCÍO ESCOBAR-BRAVO ‡**

Research group Plant Ecology and Phytochemistry, Cluster Plant Sciences and Natural Products, Institute of Biology, Leiden University, The Netherlands

***** Corresponding author

‡ Shared last co-authorship

**Current address: Institute of Plant Sciences, University of Bern, Altenbergrain 21, 3013 Bern, Switzerland

Gang Chen, email: g.chen@sicau.edu.cn

Supplementary materials

Table S1 Breeding IDs of the 95 chrysanthemum cultivars used in this study

Cultivar	Breeding ID	Cultivar	Breeding ID	Cultivar	Breeding ID	Cultivar	Breeding ID
1	DC-1	25	DC-25	49	48837	73	56072
2	DC-2	26	DC-26	50	48639	74	56168
3	DC-3	27	DC-27	51	9403	75	56701
4	DC-4	28	DC-28	52	41475	76	56703
5	DC-5	29	26741	53	45644	77	56713
6	DC-6	30	31563	54	45785	78	56817
7	DC-7	31	8713	55	48286	79	57352
8	DC-8	32	7688	56	40931	80	57709
9	DC-9	33	30600	57	43339	81	57773
10	DC-10	34	21697	58	90753	82	57993
11	DC-11	35	13185	59	36318	83	58498
12	DC-12	36	8578	60	43110	84	59209
13	DC-13	37	8393	61	44339	85	64952
14	DC-14	38	4875	62	48942	86	65001
15	DC-15	39	48864	63	9361	87	37511
16	DC-16	40	47287	64	42215	88	37577
17	DC-17	41	57067	65	42377	89	37630
18	DC-18	42	55229	66	42629	90	42415
19	DC-19	43	55238	67	42909	91	25533
20	DC-20	44	46885	68	50223	92	22898
21	DC-21	45	55223	69	50858	93	48015
22	DC-22	46	55115	70	51643	94	20880
23	DC-23	47	45728	71	56068	95	49230
24	DC-24	48	90633	72	56069		

Table S2 Detailed statistical analysis performed for data displayed in each figure

**Figure**	**Panel**	**Statistical test**	**Factor and statistic value**	**Degree of freedom**	**Significance**
Fig. 2	a	ANOVA	Genotype; $F = 6.59$	$df1 = 94$, $df2 = 190$	$\boldsymbol{P < 0.001}$
	b	Kruskal-Wallis	Genotype; $\chi^2 = 250.1$	$df = 94$	$\boldsymbol{P < 0.001}$
	c	Spearman correlation	$r = 0.269$; $N = 95$	-	$\boldsymbol{P = 0.008}$
Fig. 3	a	Pearson correlation	$r = 0.186$, $N = 12$	-	$P = 0.564$
	b	Pearson correlation	$r = 0.118$; $N = 12$	-	$P = 0.715$
	c	Pearson correlation	$r = 0.293$; $N = 12$	-	$P = 0.355$
Fig. 4	a	GLM	Genotype; *Wald* $\chi^2 = 70.979$	$df = 5$	$\boldsymbol{P < 0.001}$
			JA or Mock; *Wald* $\chi^2 = 111.912$	$df = 1$	$\boldsymbol{P < 0.001}$
			Interaction; *Wald* $\chi^2 = 33.160$	$df = 5$	$\boldsymbol{P < 0.001}$
	b	GLM	Genotype; *Wald* $\chi^2 = 58.106$	$df = 5$	$\boldsymbol{P < 0.001}$
			JA or Mock; *Wald* $\chi^2 = 195.343$	$df = 1$	$\boldsymbol{P < 0.001}$
			Interaction; *Wald* $\chi^2 = 25.740$	$df = 5$	$\boldsymbol{P < 0.001}$
	c	GLM	Genotype; *Wald* $\chi^2 = 261.895$	$df = 5$	$\boldsymbol{P < 0.001}$
			JA or Mock; *Wald* $\chi^2 = 5.034$	$df = 1$	$\boldsymbol{P = 0.025}$
			Interaction; *Wald* $\chi^2 = 12.661$	$df = 5$	$\boldsymbol{P = 0.027}$
	d	GLM	Genotype; *Wald* $\chi^2 = 1036.121$	$df = 5$	$\boldsymbol{P < 0.001}$
			JA or Mock; *Wald* $\chi^2 = 0.010$	$df = 1$	$P = 0.922$
			Interaction; *Wald* $\chi^2 = 4.057$	$df = 5$	$P = 541$
Fig. 5	a	Pearson correlation	$r = -0.619$; $N = 6$	-	$P = 0.190$
	b	Pearson correlation	$r = -0.118$; $N = 6$	-	$P = 0.824$
	c	Pearson correlation	$r = -0.037$; $N = 6$	-	$P = 0.944$
Fig. S1	a	Kruskal-Wallis	Genotype; $\chi^2 = 48.8$	$df = 11$	$\boldsymbol{P < 0.001}$
	b	ANOVA	Genotype; $F = 3.32$	$df1 = 11$, $df2 = 48$	$\boldsymbol{P = 0.002}$
Fig. S2	a	Pearson correlation	$r = -0.376$; $N = 12$	-	$P = 0.228$
	b	Pearson correlation	$r = -0.567$; $N = 12$	-	$P = 0.055$

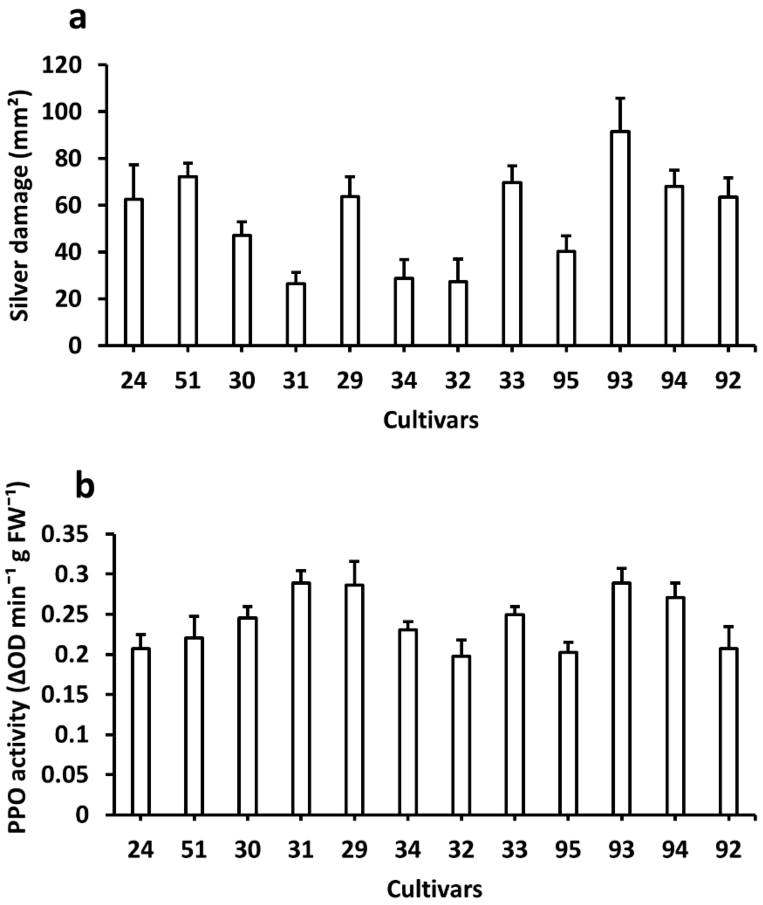

Fig. S1 Phenotypic variation in Western flower thrips resistance and polyphenol oxidase activity among chrysanthemum cultivars. (a) Silver damage symptoms (mean ± SEM, n = 10) and (b) polyphenol oxidase (PPO) activity (mean ± SEM, n = 5) were determined in 12 different chrysanthemum cultivars. Plants were sampled for PPO activity measurement or used for non-choice whole plant bioassays at 35 days after planting. Western flower thrips (WFT) leaf damage ('silver damage') was determined at 7 days after WFT infestation

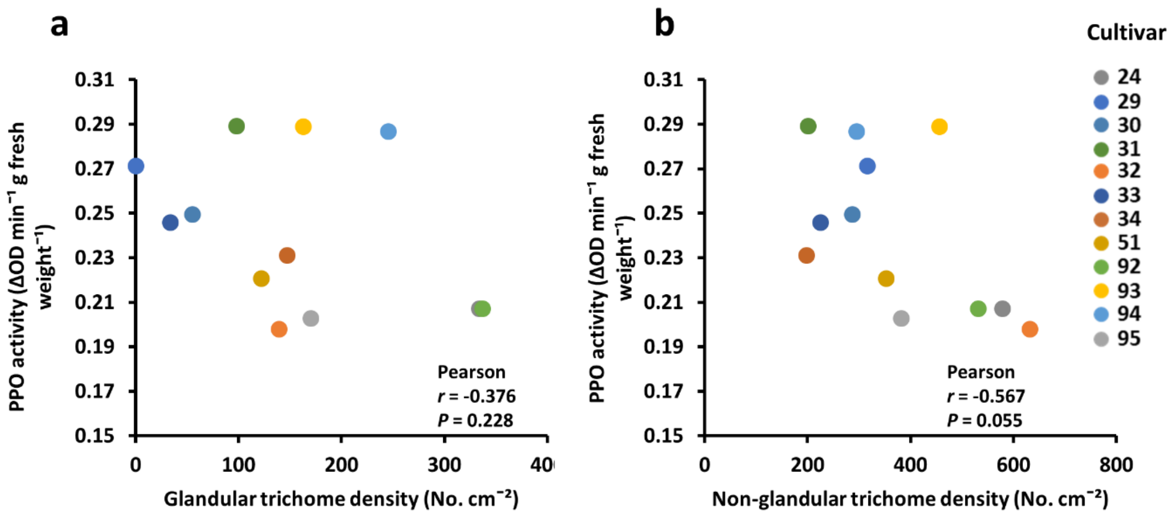

Fig. S2 Relationship between polyphenol oxidase activity and glandular/non-glandular trichome density in chrysanthemum. Scatter plots depicting the relationship between (a) polyphenol oxidase (PPO) activity and glandular trichome density, and (b) PPO activity and non-glandular trichome density. Plants were sampled for PPO activity and trichome density at 35 days after planting. The plots display data obtained from 12 chrysanthemum cultivars. Each dot corresponds to the mean of five plant replicates per cultivar for PPO and trichome density, and of ten plant replicates per cultivar for silver damage symptoms
